# Why are we not flooded by involuntary thoughts about the past and future? Testing the cognitive inhibition dependency hypothesis

**DOI:** 10.1007/s00426-018-1120-6

**Published:** 2018-11-27

**Authors:** Krystian Barzykowski, Rémi Radel, Agnieszka Niedźwieńska, Lia Kvavilashvili

**Affiliations:** 1 Applied Memory Research Laboratory, Institute of Psychology, Jagiellonian University, ul. Ingardena 6, 30-060 Kraków, Poland; 2 Université Côte d’Azur, Nice, France; 3 University of Hertfordshire, Hatfield, UK

**Keywords:** Involuntary memories, Involuntary future thoughts, Autobiographical memory, Inhibition, Cognitive control, Mental time travel

## Abstract

In everyday life, involuntary thoughts about future plans and events occur as often as involuntary thoughts about the past. However, compared to involuntary autobiographical memories (IAMs), such episodic involuntary future thoughts (IFTs) have become a focus of study only recently. The aim of the present investigation was to examine why we are not constantly flooded by IFTs and IAMs given that they are often triggered by incidental cues while performing undemanding activities. One possibility is that activated thoughts are suppressed by the inhibitory control mechanism, and therefore depleting inhibitory control should enhance the frequency of both IFTs and IAMs. We report an experiment with a between-subjects design, in which participants in the depleted inhibition condition performed a 60-min high-conflict Stroop task before completing a laboratory vigilance task measuring the frequency of IFTs and IAMs. Participants in the intact inhibition condition performed a version of the Stroop task that did not deplete inhibitory control. To control for physical and mental fatigue resulting from performing the 60-min Stroop tasks in experimental conditions, participants in the control condition completed only the vigilance task. Contrary to predictions, the number of IFTs and IAMs reported during the vigilance task, using the probe-caught method, did not differ across conditions. However, manipulation checks showed that participants’ inhibitory resources were reduced in the depleted inhibition condition, and participants were more tired in the experimental than in the control conditions. These initial findings suggest that neither inhibitory control nor physical and mental fatigue affect the frequency of IFTs and IAMs.

**Electronic supplementary material:**

The online version of this article (10.1007/s00426-018-1120-6) contains supplementary material, which is available to authorized users.

## Introduction

Every time we project ourselves back in time to re-experience personal past, or forward in time to think about possible future events, we engage in episodic mental time travel (MTT; Suddendorf, Addis, & Corballis, 2009). Past research has focused predominantly on deliberate forms of MTT (Wheeler, Stuss, & Tulving, 1997; D’Argembeau & Van der Linden, 2004). For example, when trying to recall the last time we ate pizza or planning our next summer holiday, we are engaged in voluntary MTT. It involves strategic and constructive retrieval or simulation of events from our episodic memory. According to the constructive episodic simulation hypothesis, thinking about the future may be even more effortful than recalling the past, because it involves a flexible re-combination of available episodic information into a novel event (Schacter and Addis 2007).

However, in everyday life, such episodic past or future-oriented thoughts may also come to mind unexpectedly without trying to think about them (e.g., Berntsen & Jacobsen, 2008). While involuntary thoughts about the past (i.e., involuntary autobiographical memories, IAMs) have been investigated extensively over the past decade, research on their prospective counterpart (i.e., episodic involuntary future thoughts, IFTs), is in its infancy with only a handful of published studies (for a review, see Berntsen 2018, this volume).

In line with research on voluntary forms of MTT, initial studies comparing involuntary episodic past and future thinking have revealed interesting similarities between the two. For example, the diary studies by Berntsen & Jacobsen (2008) and Finnbogadóttir & Berntsen (2013) showed that in everyday life, IFTs occur as frequently as IAMs, suggesting that involuntary occurrence of such thoughts could be the basic mode in which our cognitive system prefers to operate in daily life (cf. Berntsen, 2010). Similar to IAMs, IFTs are more likely to occur when an individual is engaged in an automatic activity with low attention demands, and in response to incidental external and internal cues that usually overlap with key features of the IFT content (e.g., when seeing a manuscript on a desk, imagining how relieved one would be to get it published one day) (Berntsen and Jacobsen, 2008; Finnbogadóttir and Berntsen, 2013).

More recently, researchers have started to investigate IFTs with a laboratory method, which was developed initially by Schlagman & Kvavilashvili (2008) to study IAMs. In these studies (Cole, Staugaard, & Berntsen, 2016; Plimpton, Patel, & Kvavilashvili, 2015; Vannucci, Pelagatti, & Marchetti, 2017), participants reported IFTs and IAMs that occurred during a vigilance task that required little attention (detecting infrequent target vertical lines in a stream of slides with horizontal lines). In addition, participants were exposed to brief (and irrelevant) verbal phrases on the screen, some of which might incidentally trigger IFTs. These studies have resulted in several additional findings demonstrating important similarities between involuntary thoughts about the past and future. For example, like IAMs, IFTs were often reported to occur in response to irrelevant word cues on the screen. They were also as specific and reported as quickly in response to word cues as IAMs (Cole, Staugaard, & Berntsen, 2016), suggesting that IFTs may rely on a similar automatic spreading activation mechanism, even though they involve imagining events that have not yet happened. Therefore, similar to IAMs, IFTs may require little effort and constructive processes. However, some differences between IAMs and IFTs were also reported. For example, more IAMs than IFTs were reported in the standard vigilance task with verbal cues (Cole et al., 2016; Plimpton et al., 2015; Vannucci et al., 2017), while this pattern was reversed in the vigilance task with no verbal cues (Vannucci et al., 2017), suggesting that in the absence of meaningful (verbal) stimuli, participants were more likely to spontaneously think about the future than the past.

This finding is particularly interesting in light of the prospective bias in task-unrelated thoughts, reported in mind-wandering research (Smallwood, Nind, & O’Connor, 2009; Stawarczyk, 2018), which has used similar vigilance and go/no-go tasks, but often without any irrelevant verbal cues (Smallwood & Schooler, 2006, 2015). It raises an interesting possibility that IFTs and IAMs may constitute the content of at least some of the task-unrelated thoughts studied in mind-wandering research (for similar views see Johannessen & Berntsen, 2010; Plimpton et al., 2015), and emphasises the importance of cross-talk between currently separate areas of research on mind-wandering and involuntary episodic past and future thinking.

## Role of cognitive inhibition in experiencing involuntary mental time travel

Although there is widespread agreement that IFTs and IAMs come to mind automatically, very little is known about their underlying cognitive mechanisms in terms of cognitive processes that enable the experience of such thoughts when one is engaged in unrelated activities (Berntsen, 2009, p. 86). The issue of why we involuntarily project ourselves back or forward in time pertains to a broader question about why are we not constantly flooded by these thoughts in daily life, given that both IFTs and IAMs are triggered automatically in response to incidental external and internal cues (Vannucci, Pelagatti, Hanczakowski, Mazzoni, & Paccani 2015). It is intriguing to ask what keeps these spontaneous mental occurrences at bay and enables us to carry on with our daily activities uninterrupted. The present paper aimed to verify one possible answer to this question, namely, that cognitive inhibition may preclude the occurrence of IFTs and IAMs when people are engaged in other activities. If there is indeed a special inhibitory mechanism that keeps spontaneously occurring past and future thoughts at bay, then they should be reported more frequently in conditions where this inhibitory mechanism is impaired compared to conditions when inhibitory control works optimally. In the present paper, we call this approach the cognitive inhibition dependency view, which is based on the model of autobiographical memory proposed by Conway et al.

According to the self-memory system model proposed by Conway & Pleydell-Pearce (2000, for later modifications see Conway, 2008, 2009; Conway & Jobson, 2012), autobiographical memory consists of a hierarchical network of interconnected nodes that differ in terms of their level of specificity. At the bottom of the network are stored fragments of events with specific sensory details (e.g., *details experienced when riding a horse for the first time*). Such vivid information is further connected to super-ordinate levels of general events (e.g., *riding a horse on Sundays*), common themes (*riding, doing sports*), or even, at the highest level of memory nodes, important periods in one’s life (e.g., *when I was still young and fit*). According to this model, the activation of autobiographical information may spread across the network resulting in the construction of a particular memory. Importantly, such activation may be elicited by different types of cues (i.e., internal or external). While voluntary autobiographical memories are the result of a top-down search process that eventually arrives at an episode, directly retrieved (i.e., involuntary) memories are thought to circumvent the search process and arrive at the episode and enter consciousness very quickly. As Conway & Pleydell-Pearce (2000) argued, fragments of memory representations are constantly activated at the bottom level of the hierarchy by different internal and external cues, but the vast majority of such memories never reach consciousness due to being suppressed by the constantly operating executive control system. Only some of these activated memories, especially those that are consistent with current self-goals, may occasionally get through this inhibitory control mechanism and reach awareness. This implies that IAMs can occur as a result of two concurrent processes, namely spreading activation (i.e., boosting the chances of memory retrieval) and inhibition processes that, in fact, may work against each other (see Ball & Hennessey, 2009). In this sense, the retrieval of IAMs would depend mainly on inefficient functioning of the inhibitory control mechanism.

Although Conway et al.’s model concerns the retrieval of autobiographical memories, it may also have interesting implications for the occurrence of IFTs in daily life. For example, there is growing evidence to show that involuntary and voluntary future thoughts are often goal-oriented, which has resulted in the use of a new term ‘autobiographical planning’ in the literature (e.g., Baird, Smallwood, & Schooler, 2011; Spreng, Gerlach, Turner, & Schacter, 2015). A recent study by Cole & Berntsen (2016) showed that IFTs were more likely to reflect people’s current concerns and goals than memories about the past (see also Anderson & McDaniel, 2018; Plimpton et al., 2015). Therefore, the occurrence of IFTs could be even more dependent on the malfunctioning of the inhibitory control mechanism specified by the self-memory system than the retrieval of IAMs.

However, the occurrence of IFTs and IAMs may also be explained within a broader framework of inhibitory control proposed by Hasher and colleagues (see Hasher, Lustig, & Zacks, 2007, 1999). According to Hasher et al. (1999), inhibition serves at least two important, but different functions: (1) suppression of automatic responses, and (2) suppression of interference from irrelevant information (see also Friedman & Miyake, 2004). While deficits in the former may result in impulsive behaviour (Nigg, 2000), deficits in the latter may result in experiencing involuntary mental contents such as memories and future thoughts in response to irrelevant stimuli or distracters in one’s environment (Dempster, 1992). Therefore, according to this view, depletion of inhibitory control resources would increase individual’s susceptibility to irrelevant stimuli in general and would result in increased likelihood of experiencing both IFTs and IAMs.

So far, to the best of the authors’ knowledge, no previous study has directly investigated the role of cognitive inhibition in the frequency of experiencing IFTs and IAMs by manipulating the levels of inhibitory control in participants. However, a couple of studies have used correlational methods to address this issue. For example, Kamiya (2014) reported a significant positive correlation (*r* (22) = 0.60, *p* < 0.01) between the number of IAMs reported during a walk around campus and the scores on the Cognitive Failures Questionnaire (CFQ, Broadbent, Cooper, FitzGerald and Parkes, 1982). The CFQ measures the frequency of minor absent-minded slips and errors in everyday life in the cognitive domains of perception, memory and action, which may reflect indirectly the weak functioning of the inhibitory processes (Friedman and Miyake, 2004). In addition, several studies on individuals with attentional deficit hyperactivity disorder (ADHD), which is characterized by low levels of inhibitory control, have demonstrated that they have elevated levels of spontaneous mind-wandering (e.g., Seli, Smallwood, Cheyne, & Smilek, 2015; see also; Jonkman, Markus, Franklin, & Van Dalfsen, 2017; Shaw & Giambra, 1993).

## The present investigation

According to the two influential theoretical models of Conway & Pleydell-Pearce (2001) and Hasher et al. (1999), the occurrence of IFTs and IAMs may depend on the intact inhibitory control mechanism that prevents our stream of consciousness from being flooded by task-unrelated thoughts about the past and future. The main aim of the present investigation was to test this assumption by experimentally depleting the inhibitory resources of participants by engaging them in cognitive tasks that require high levels of inhibitory control. We based this manipulation on the idea that inhibition relies on limited resources that cannot be continuously maintained for a long time and thus are easily depleted (Muraven & Baumeister, 2000; Schmeichel, 2007). For example, participants in a recent study by Radel, Davranche, Fournier, & Dietrich (2015) performed a high conflict task (the Simon task^1^ in Study 1a and 2, and the Eriksen Flanker task^2^ in Study 1b) for 40 min in which 50% of trials were incongruent trials. In such conflict tasks, participants can accurately respond to a target stimulus only by selecting its relevant feature and by inhibiting the irrelevant feature that automatically triggers the incorrect response. While in the congruent trials, both the irrelevant information (e.g., the flankers) and the relevant information (e.g., the centre target arrow) are mapped to the same responses (e.g., are pointing in the same direction), in the incongruent trials they are associated with different responses. As a result, reaction times and performance accuracy are usually reported to be better for congruent than for incongruent trials. This interference effect (measured by subtracting reaction times of incongruent and congruent trials) is considered as a reliable indicator of the cognitive control efficacy (e.g., Van Den Wildenberg, Wylie, Forstmann, Burle, Hasbroucq, & Ridderinkhof, 2010). Radel et al. (2015) showed that only participants engaged in the high interference version of a conflict task had impaired cognitive resources for inhibition, providing strong support to the idea that inhibitory resources are indeed limited and can be easily depleted by a simple experimental manipulation (see also, Schmeichel, 2007).

In the present study, to test the cognitive inhibition dependency hypothesis, we depleted the cognitive control prior to measuring the frequency of IFTs and IAMs in the standard vigilance task (e.g., Plimpton et al., 2015). More specifically, we engaged participants in the prolonged practice of a cognitive task that differed in the amount of inhibitory control required. We used a well-known Stroop conflict task (Stroop, 1935; Kane & Eagle, 2003) where participants have to react as fast as possible in response to the colour of the ink (e.g., red) of a given word (e.g., ‘blue’) while ignoring its meaning (‘blue’). The main differences between the current procedure and the Radel et al.’s (2015) original manipulation were as follows: (1) use of the Stroop task to deplete inhibition, (2) extending the manipulation from 40 to 60 min, (3) increasing the number of incongruent trials in the depleted inhibition condition from 50 to 75%. In the intact depletion condition, participants named the colour of the ink, which was identical to the meaning of the word (e.g., word ‘red’ printed in red ink) for 60 min. Importantly, we controlled the level of tiredness and fatigue in these two experimental conditions by having the control group of participants who were not engaged in the Stroop task before the vigilance task.

To measure the frequency of IFTs and IAMs under well-controlled laboratory conditions, participants were engaged in a vigilance task in which they had to detect infrequent target slides with vertical lines and ignore the non-target slides with patterns of horizontal lines (see Plimpton et al., 2015). In addition, they were exposed to short verbal phrases, some of which could incidentally trigger task-unrelated thoughts, including IFTs and IAMs. Throughout the vigilance task, participants were probed at random intervals to record their thoughts at the moment they were stopped. Finally, on completion of the task, participants were given their thought descriptions and were asked to indicate whether they were memories or future thoughts.

According to the cognitive inhibition dependency view, the frequency of IFTs and IAMs should be influenced by the level of available inhibitory resources. Thus, we expected to observe higher frequencies of IFTs and IAMs when inhibitory resources were depleted, namely, after performing the incongruent Stroop task compared to when these resources were not depleted (i.e., after performing the congruent Stroop task). Given that IFTs seem to be more consistent with one’s current goals and concerns than IAMs (Cole & Berntsen, 2016), it is also possible to observe more IFTs than IAMs in the depleted inhibition condition compared to the intact inhibition and control conditions. Alternatively, if such involuntary cognitions are not dependent on the levels of available inhibitory control, the experimental and control conditions should not differ in the frequency of IFTs and IAMs.

## Method

### Design

A mixed subjects design was employed in the present study. The between-subjects variable was the levels of available cognitive inhibitory resources just before the vigilance task (depleted inhibition, intact inhibition, control group), and the within-subjects variable was the type of involuntary thought reported during the vigilance task (IFTs, IAMs).

### Participants

The Research Ethics Committee approved the Study. Written consent for participation was obtained prior to data collection. Importantly, during the recruitment process, all participants were clearly informed that the laboratory session was very demanding and involved performing attentionally demanding tasks for prolonged periods of time. They were informed that they were free to withdraw from the study at any point.

A total of 124 participants (85 females, *M*_age_ = 22.61, SD = 2.51, range 19–37 years) were recruited and randomly assigned to the three experimental conditions: the control, the depleted inhibition and the intact inhibition conditions. Participants were tested in groups of two to eleven in a laboratory with separate computer stations.

Nine participants guessed the true purpose of the study, and their results were excluded from the analysis concerning the frequency of involuntary task-unrelated thoughts, including IFTs and IAMs. Due to technical difficulties, one additional participant did not finish the experiment. Therefore, the final sample consisted of 114 participants with 38 participants in the control condition (27 females, *M*_age_ = 22.95, SD = 2.91, range 20–37 years), 38 participants in the intact inhibition condition (25 females, *M*_age_ = 22.25, SD = 1.85, range 19–28 years) and 38 participants in the depleted inhibition condition (25 females, *M*_age_ = 22.20, SD = 1.63, range 19–25 years). Students participated in return for a 50 PLN (ca. 14 USD).

### Materials

#### The vigilance task

In the present study, we used a fully computerized version of the vigilance task described elsewhere in more detail (e.g., Barzykowski & Niedźwieńska, 2016, pp. 5–6; also; Barzykowski & Staugaard, 2016, p. 524), which was very similar to the procedure used by Plimpton et al. (2015) to study IAMs and IFTs under laboratory conditions (adapted from Schlagman & Kvavilashvili, 2008). The main differences between the present task and Plimpton et al.’s (2015) design were as follows: (1) using 400 slides instead of 600, (2) random presentation of trials for each participant, (3) extending the presentation of each trial from 1.5 to 2.5 s,^3^ (4) providing participants with 12 fixed stops with thought probes, and (5) participants typing their answers to thought questionnaires.

The vigilance task involved detecting patterns of vertical lines (eight target slides) in a stream of 392 non-target slides with horizontal lines. Slides were presented for 2.5 s with short verbal phrases (e.g., *driving a car, swimming in the sea*) displayed in the centre of each slide. There were approximately equal numbers of neutral (*N* = 134), positive (*N* = 133), and negative (*N* = 133) phrases, that constituted the final pool of 400 phrases, which were randomly selected from the pool of 800 phrases used in previous studies (e.g., Barzykowski & Niedźwieńska, 2016, 2018a; Barzykowski & Staugaard, 2016, 2018).^4^ While the random selection of word phrases from the pool was exactly the same for all participants, the order was randomized for each participant. The program stopped automatically at 12 fixed points during the presentation with the following message appearing on the screen *“Please stop and record your concentration and thoughts now*”. Participants provided a brief description of the content of their thoughts (by typing it into the computer program), indicated how much they were concentrating on the task when stopped (1 = *Not at all*; 7 = *Fully concentrating*) and if the thought occurred deliberately (they decided to think about it) or involuntarily (it simply popped in their mind). The probes, which were modelled on previous literature (Plimpton et al., 2015), were presented in a fixed pseudo-random order and occurred at varying intervals consisting of a minimum of 22 (about 55 s) and a maximum of 42 (about 105 s) slides. Importantly, these intervals between the stops were comparable to those reported by Plimpton et al. (2015), which varied between 52.5 and 105 s.

#### Experimental manipulation of inhibitory resources (the Stroop task)

Two versions of the Stroop-like task were used to manipulate the availability of resources for cognitive inhibition before starting the vigilance task (MacLeod, 1991; Chuderski, Taraday, Nęcka, & Smoleń, 2012). This task consisted of four colour words (red, green, blue, and yellow) in Polish, printed in one of these four colours (e.g., the word red could be printed in red, green, blue or yellow colours). Participants were instructed to judge the colour of the ink of the word as fast as possible, without paying attention to the meaning of the word, by pressing a key corresponding to the colour of the ink. Each word was displayed for 2000 ms with 150 ms interval between trials and a 1000 ms feedback screen after each trial. While the meaning of the word and the colour of the ink were the same in congruent trials, the meaning differed from the colour of the ink in incongruent trials. In the inhibition depletion condition, 75% of trials were incongruent. In the intact inhibition condition, 100% of trials were congruent. Both of these tasks lasted 60 min and were presented on a computer using E-prime 2 (PST, Pittsburgh, USA). Finally, for a practice trial, we used a short 2-min version of the Stroop task that consisted of 50% congruent and 50% incongruent trials.

#### Manipulation checks for depleting inhibitory resources (the Simon task)

We used the Simon task (Simon, 1990) to assess the impact of manipulating inhibitory control resources by measuring the inhibition performance before and after the extended completion of the Stroop-like task. The Simon task consists of a yellow or pink circle appearing either on the left or on the right side of the screen. Participants were instructed to respond as fast as possible by pressing a left (S) or right (L) key of the keyboard with the left or right index finger according to the colour of the stimulus (left-pink; right-yellow). Each stimuli appeared after a fixation cross presented at the centre of the screen for 200 ms. In congruent trials, the spatial location of the stimulus and response were the same (e.g., right stimulus/right response). In the incongruent trials, the spatial location of the stimulus was opposite to the location of the response (e.g., right stimulus/left response), which triggered an automatic response that had to be inhibited. The Simon task consisted of 160 trials with 600 ms intervals between each trial. At the completion of the task, participants were provided with feedback about their performance (mean reaction time and percent of correct responses). The task lasted about 2–3 min and was presented on a computer using E-prime (PST, Pittsburgh, USA).

#### The positive and negative affect schedule (PANAS; Brzozowski, 2010)

This scale measures the strength of negative and positive emotions and consists of 30 items measuring current emotional states. Participants have to rate on a five-point scale to what extent the given adjectives correspond with their current state. The reliability coefficients (internal consistency and stability) of the Polish version of the PANAS range from 0.73 to 0.95 (Brzozowski, 2010). It was used to control for possible differences between the conditions. For example, it was possible that performing a high-conflict task (i.e., incongruent version of the Stroop task) would negatively affect participants’ mood.

### Procedure

**Fig. 1:**
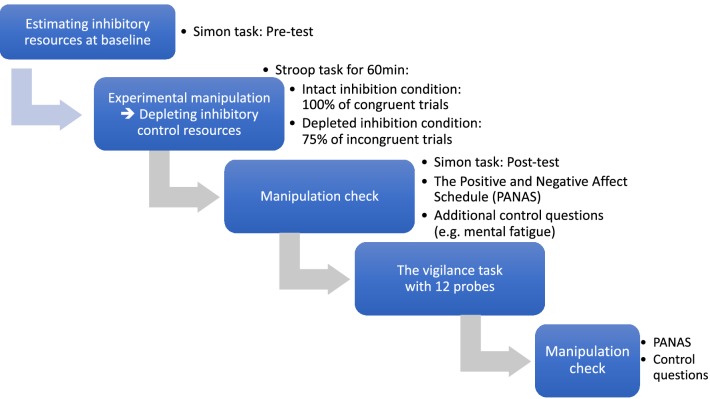
An overview of the experimental procedure. While depleted and intact inhibition conditions followed all steps, the control group performed only the vigilance task and completed the PANAS and additional control questions before and after the vigilance task

The control participants did not perform the Stroop task before the vigilance task, while participants in the other two conditions performed the incongruent and congruent versions of the Stroop task, respectively, before completing the vigilance task. An overview of the procedure is presented in Fig. 1.

#### Control condition

At the beginning, participants completed a questionnaire about the activities they were involved in before the experimental session and their current levels of fatigue. Using seven-point scales, participants rated the extent to which previously performed activities were tiring, difficult, how much concentration they required and the extent to which participants had been performing them as well as they could. Participants also rated their current level of general, physical and mental fatigue (on all scales, one corresponded to not endorsing the item at all, four corresponded to average endorsement, and seven corresponded to highly endorsing the item). All points along the scale were clearly labelled.

Then, participants were verbally informed that the experiment examined how people concentrated on monotonous and boring tasks. The experimenter only briefly introduced the participants to the procedure by providing verbal instructions about how to complete the vigilance task. Next, participants started the computerized vigilance task, which provided them with more detailed written instructions. In particular, as in previous studies (e.g., Barzykowski & Niedźwieńska, 2016, 2018a, 2018b; Barzykowski & Staugaard, 2016, 2018; Plimpton et al., 2015; Vannucci, Batool, Pelagatti, & Mazzoni, 2014, 2015), participants were instructed to identify slides with vertical lines by pressing a red button (“m” on the keyboard). In addition, they were informed that they would also see word phrases in the centre of each slide. It was explained that these word phrases were used in another condition and they should not respond to them during the current study. Next, participants were engaged in the first practice session that required responding only to target slides with vertical lines. It lasted about one minute and consisted of 25 non-target and 2 target stimuli, with no stop trials.

After the first practice task, participants were informed that during the vigilance task they might experience different kinds of task-unrelated thoughts, and they were provided with examples of such thoughts, including personal goals, words, random associations, current concerns, future thoughts, plans, and memories. However, no particular emphasis was put on memories and future-oriented thoughts during the briefing. Participants were only informed that thoughts could be diverse (i.e., specific, general) and pertain either to the present, past or future. Importantly, they were assured that these thoughts could be about anything and that they could simply pop into their mind spontaneously or they could have chosen to deliberately think about them. It was explained, that since the study was about people’s attention and concentration, the program would occasionally stop, and they would be asked to record their concentration level and the content of their thoughts at the exact moment they were stopped. Importantly, participants were encouraged to record the content of their thoughts at the exact moment they were stopped, regardless of what it was. They were also assured that their responses would be anonymous, and informed that they could refrain from reporting particularly sensitive thoughts by typing “X” as an answer, or (if possible) by providing a general description of their thoughts rather than a detailed account (see Appendix for the exact written instructions provided to participants).

Next, participants performed the second practice task that was the same as the first one with the addition of one stop trial.^5^ Once the practice task was completed, participants filled in the Positive and Negative Affect Schedule (PANAS; Brzozowski, 2010), and then started the main vigilance task. Each time the program stopped, they were asked to provide a brief description of the content of their thoughts (by typing it into the computer program), rate their level of concentration (on a seven-point scale) and indicate if the thought occurred deliberately (they decided to think about it) or involuntarily (it simply popped into their mind).

After completing the vigilance task, participants answered open-ended questions about what they thought the true goal of the study was. They were then provided briefly with verbal instructions describing the nature of autobiographical memory (as, for example, in Schlagman, Kliegel, Schulz, & Kvavilashvili, 2009, p. 410) and future thoughts and were informed about the second part of the study. During this part, the program provided participants with more detailed written instructions. Participants reviewed all of their thoughts recorded during the vigilance task, one at a time and in the same order as they had been recorded. Participants were instructed to decide whether each thought was an autobiographical memory, future-oriented thought, thought relating to the current situation or other type of thought by clicking the appropriately labelled button. Choosing the last two options displayed the next recorded thoughts. If participants chose one of the first two options, they were asked to describe the memory or future-oriented thought more thoroughly by typing it into the computer program. This way we obtained longer descriptions of these mental contents than during the vigilance task.

#### Depleted inhibition condition (the incongruent version of the Stroop task)

The only difference between the control and the depleted inhibition condition was that before the vigilance task participants performed an incongruent version of the Stroop task. As in the control condition, participants were informed that the experimental session examined how people concentrated on monotonous and boring tasks. It was explained that for this reason they would do a few vigilance tasks. While we did not explain in more detail the Simon task and Stroop-like task saying that detailed instructions would be provided in the program, we explained in more detail the vigilance task the same way as in the control conditions. Then, participants started with the first Simon task (pre-test) that allowed us to evaluate baseline inhibition performance. As in the Radel et al.’s (2015) study, to get a stable and coherent indicator of the individual’s inhibitory control, each participant did the task at least four times. If their responses in the fourth task were stabilized (accuracy and RT below 5% of variation), sufficiently fast (average RT below 700 ms) and accurate (above 80% of correct responses), the training stopped at this point, indicating that there was no further effect of learning on the task and results mainly depended on the inhibitory control. Otherwise, participants had to complete additional runs of the task until they met these criteria. Next, participants trained on the short version of the Stroop task for about 2 min and then started the 60-min version of the incongruent Stroop task. Immediately after the Stroop task, participants completed the second Simon task (post-test) which was performed only once for about 2–3 min, which allowed us to estimate the alteration of the inhibition capacity induced by the prolonged exposure to the congruent Stoop like task. Finally, just before starting the computer program with the vigilance task, participants filled in the PANAS and answered control questions concerning the activities they were just involved in and their current level of fatigue, which took no longer than 5 min.

#### Intact inhibition condition (the congruent version of the Stroop task)

The only difference between the depleted inhibition condition group and the intact inhibition condition was that participants performed the low-conflict version of the Stroop task with 100% of congruent trials (i.e., 0% of incongruent trials).

## Results

For all statistical analyses reported below, the level of significance was set at *p* < .05, and the effect size was measured by partial eta-squared (*η*_*p*_^*2*^*)*.

### Equivalence of experimental conditions before and after the vigilance task

To test the comparability of conditions before and after the vigilance task in terms of participants’ mood ratings, the overall mean positive and negative PANAS scores were entered into two separate 3 condition (control, depleted inhibition, intact inhibition) × 2 time of testing (before vs. after the vigilance task) mixed ANOVAs with repeated measures on the last factor. The analysis on the mean positive affect scores revealed a significant main effect of time of testing, *F*(1, 119) = 29.70, *p* < 0.001, *η*_*p*_^*2*^ = 0.20, with scores being significantly lower after completing the vigilance task (see Table 1). While the group by time interaction was not significant (*F* < 1), the main effect of group was approaching significance, *F*(1, 119) = 3.06, *p* = 0.050, *η*_*p*_^*2*^ = 0.05, with higher scores in the control group compared to the intact and depleted inhibition conditions (*p* = 0.015), which did not differ from each other (*p* = 0.258). For negative affect scores, none of the main or interaction effects were significant (all *F*_s_ < 1).

**Table 1:** Means and standard deviations for variables measuring mood, fatigue, type of activities performed before the vigilance task, and performance on vigilance task as a function of condition (control, intact inhibition, depleted inhibition)

	Condition					
	Control		Intact inhibition (congruent Stroop task)		Depleted inhibition (incongruent Stroop task)	
	M	SD	M	SD	M	SD
Mood ratings						
PANAS: Positive affect_1	43.88	9.24	37.46	10.74	39.63	9.99
PANAS: Positive affect_2	36.66	10.00	32.73	11.87	35.35	13.33
PANAS: Negative affect_1	20.07	6.84	19.02	5.86	18.93	5.92
PANAS: Negative affect_2	18.98	7.26	19.32	6.31	18.30	5.98
Activities before the vigilance task						
Tiring	2.83^1, 2^	1.38	5.95^1^	1.00	6.03^2^	0.92
Difficult	2.23^1, 2^	1.05	3.38^1^	1.41	3.98^2^	1.58
Concentration	3.82^1, 2^	1.38	5.36^1^	1.25	5.78^2^	1.12
General motivation to perform these activities well	4.98^1, 2^	1.33	6.31^1^	0.73	6.25^2^	0.71
General fatigue	2.63^1, 2^	1.43	5.18^1^	1.21	5.40^2^	1.28
Physical fatigue	3.15^1^	1.51	4.08^1^	1.55	3.53	1.50
Mental fatigue	2.90^1, 2^	1.19	5.03^1^	1.18	4.98^2^	1.40
After the vigilance task						
How tiring was the vigilance task	3.25	1.39	3.64	1.69	4.13	1.83
Performance on vigilance task						
Proportion of targets detected	0.93	0.14	0.88	0.18	0.89	0.15
Response time (in seconds)	0.77^1, 2^	0.16	0.95^1^	0.26	0.90^2^	0.22
Concentration rating	4.62^1, 2^	1.09	3.37^1^	1.26	3.59^2^	1.02

All questions but PANAS were rated on 7-point scales (1 = low to 7 = high). Means with the same numerical subscripts (e.g., 1, 2) are significantly different between columns. Please note that, while Stroop task in the intact inhibition condition consisted only of congruent trials, in the depleted inhibition condition it consisted of 75% of incongruent trials

Table 1 also shows mean ratings of various cognitive and non-cognitive variables provided by participants either before or after completing the vigilance task. A series of one-way ANOVAs on these means with the condition (control, depleted inhibition, intact inhibition) as a between-subjects variable, resulted in statistically significant main effects for all the variables concerning the activity or tasks performed before the vigilance task (in the intact and depleted inhibition conditions the ratings were made for the Stroop task). Specifically, significant main effects emerged for how tired participants felt [*F*(2, 116) = 106.53, *p* < 0.001, *η*_*p*_^*2*^ = 0.65], how difficult the tasks were [*F*(2, 116) = 17.08, *p* < 0.001, *η*_*p*_^*2*^ = 0.23], levels of concentration [*F*(2, 116) = 26.89, *p* < 0.001, *η*_*p*_^*2*^ = 0.32], general motivation to perform the tasks well [*F*(2, 116) = 24.09, *p* < 0.001, *η*_*p*_^*2*^ = 0.29], general fatigue [*F*(2, 116) = 55.37, *p* < 0.001, *η*_*p*_^*2*^ = 0.49], mental fatigue [*F*(2, 116) = 36.62, *p* < 0.001, *η*_*p*_^*2*^ = 0.39], and physical fatigue [*F*(2, 116) = 3.71, *p* = 0.027, *η*_*p*_^*2*^ = 0.06].

Post hoc tests indicated that participants in the control condition rated the activities performed before the vigilance task as less tiring, less difficult and requiring less concentration than participants in the depleted and intact inhibition conditions (all *p* < 0.001). In addition, they were less motivated to perform these activities as well as possible, compared to other two experimental conditions, and they were reporting lower levels of general fatigue, and psychological fatigue before commencing the vigilance task (all *p* < 0.001). Participants in the control condition were less physically tired only compared to the intact inhibition condition (*p* = 0.008). No statistically significant differences (all *p* > 0.06) were obtained between the depleted and intact inhibition conditions, which means that any differences in the number of involuntary memories and future thoughts between these conditions should not be due to differences in any of the variables listed above. Finally, a one-way ANOVA on ratings of how tiring participants found the vigilance task, did not result in a significant main effect of condition [*F*(2, 116) = 2.83, *p* = 0.063, *η*_*p*_^*2*^ = 0.05).

### Manipulation checks (the Simon task)

The performance on the Simon task was analyzed to examine if our experimental manipulation to deplete inhibitory control in the depleted inhibition condition by the incongruous Stroop task was successful. Response times of correct responses (RT) and accuracy (ACC, percentage of correct responses) were obtained after excluding the first trial of each Simon task, because these trials could be affected by participants’ initial position adjustments. In addition, abnormally short (below 100 ms) and long (above 1300 ms) responses that typically correspond to anticipations and unattended responses were excluded.^6^ Descriptive statistics for response times and accuracy scores are presented in Table 2 .

**Table 2:** Means and standard deviations for reaction times and accuracy measured in Simon task before and after prolonged exposure to incongruent Stroop task (depleted inhibition condition) and congruent Stroop task (intact inhibition condition)

Time of measurement	Condition	Congruence	Mean	SD	Mean	SD
Before	Depleted inhibition	Congruent trials	458	132	94.8	22.3
		Incongruent trials	476	122	91.2	28.3
	Intact inhibition	Congruent trials	446	106	94.9	22.0
		Incongruent trials	469	107	92.8	25.9
After	Depleted inhibition	Congruent trials	465	133	92.6	26.2
		Incongruent trials	489	139	88.8	31.6
	Intact inhibition	Congruent trials	461	117	95.2	21.3
		Incongruent trials	483	114	91.7	27.5

Mean response times were entered into a two times (before vs. after the Stroop task) x type of trials in the Simon task (congruent vs. incongruent) as within-subject factors and the condition (depleted vs. intact inhibition) as a between-subjects factor. A significant main effect of the time of measurement was found indicating longer RT after (*M* = 474 ms, SE = 6 ms) than before (*M* = 461 ms, SE = 5 ms) the Stroop task, *F*(1, 80) = 12.16, *p* = 0.001, *η*_*p*_^*2*^ = 0.13. A significant effect of congruence was also found indicating longer RT for incongruent (*M* = 478 ms, SE = 5 ms) than congruent (*M* = 457 ms, SE = 5 ms) trials, *F*(1,80) = 111.45, *p* < 0.001, *η*_*p*_^*2*^ = 0.58. None of the two-way interactions were significant (*F*s < 1). Critically, however, a three-way interaction between the time of measurement, type of trial in the Simon task, and the condition was found, *F*(1,80) = 4.85, *p* = 0.031, *η*_*p*_^*2*^ = 0.06.

**Fig. 2:**
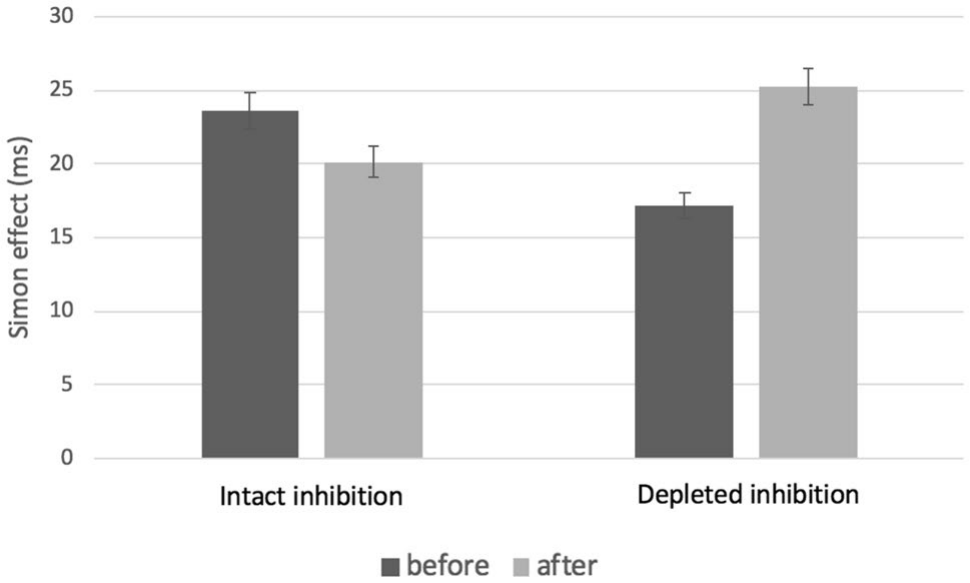
The mean reaction times in Simon task before and after prolonged exposure on the incongruent Stroop task (depleted inhibition condition) and congruent Stroop task (intact inhibition). The Simon effect scores were calculated as subtracting RTs on congruent trials from RTs on incongruent trials. Error bars indicate 95% confidence intervals for the comparison groups

This effect can be best teased apart by examining the standard measure of the Simon effect, i.e., the difference between the mean response times of incongruent and congruent trials, which represents the time needed to inhibit the interference (Simon, 1990). A mixed two (condition) by two (time of measurement) ANOVA on these response time difference scores resulted in a significant interaction between these variables, *F*(1, 80) = 4.85, *p* = 0.031, *η*_*p*_^*2*^ = 0.06, indicating that while participants in the intact inhibition condition took a similar amount of time to inhibit the interference before and after the Stroop task (*t*(40) = 0.982, *p* = 0.332), participants in the depleted inhibition condition were slower to exert inhibition after than before the Stroop task (*t*(40) = 2.081, *p* = 0.044) (see Fig. 2). This effect cannot be explained by a pre-existing group difference because no between-group difference was present at baseline, [*F*(1, 80) = 1.81, *p* = 0.182, *η*_*p*_^*2*^ = 0.02]. Similarly, no differences between the groups were present at baseline in response times to congruent and incongruent trials separately, *F*(1, 80) = 1.58, *p* = 0.212, *η*_*p*_^*2*^ = 0.02 and *F*(1, 80) = 0.41, *p* = 0.53, *η*_*p*_^*2*^ = 0.005, respectively.

The two (condition) by two (trial congruence) by two (time of measurement) mixed ANOVA on accuracy scores resulted only in significant main effects of time of measurement (*F*(1,80) = 7.23, *p* = 0.009, *η*_*p*_^*2*^ = 0.08), and congruence (*F*(1,80) = 49.296, *p* < 0.001, *η*_*p*_^*2*^ = 0.38), with more correct responses after (*M* = 93.4%, SE = 0.5%) than before (*M* = 91.9%, SE = 0.7%) the Stroop task, and less correct responses for the incongruent (*M* = 91.0, SE = 0.7%) than congruent trials (*M* = 94.3, SE = 0.4%). Because no main effect of condition or interaction effects were significant (ps > 0.095), the differences found between the two conditions in terms of response times in the previous analysis cannot likely be explained in terms of a possible change in the speed–accuracy trade-off. To completely rule out this possibility, a composite index (also called throughput rate) was created by calculating the ratio of accuracy over time (RT/accuracy ratio), representing the processing efficiency (Salthouse & Hedden, 2010). This composite index was entered into a mixed two (condition) by two (trial congruence) by two (time of measurement) ANOVA, which also revealed no main or interaction effect of the condition on processing efficiency (ps > 0.116).

In sum, this pattern of findings suggests that the manipulation of levels of inhibition in the Stroop task was effective in depleting inhibitory resources in participants who performed the incongruent Stroop task, but not in participants who performed the congruent Stroop task (i.e., depleted and intact inhibition conditions, respectively).

### Performance on the vigilance task

All participants successfully completed the vigilance task. The results are presented in Table 1. While there were no significant differences between the conditions in terms of the proportion of targets detected [*F*(2, 111) = 0.97, *p* = 0.384, *η*_*p*_^*2*^ = 0.02], there were significant main effects for response times to targets [*F*(2, 109) = 6.22, *p* = 0.003, *η*_*p*_^*2*^ = 0.10] and levels of concentration reported [*F*(2, 111) = 13.32, *p* < 0.001, *η*_*p*_^*2*^ = 0.19]. Specifically, participants in the control condition were faster at responding to targets than participants in the intact and depleted conditions (*p* = 0.001 and *p* = 0.013, respectively), and reported higher levels of concentration on the vigilance task than participants in the two other conditions (*p* < 0.001 for both conditions), which did not differ from each other on these variables (*F* < 1).

### Frequency and type of recorded thoughts

Given that each participant was stopped 12 times during the vigilance task, the total number of thought probes was 456 (12 × 38) in each condition. On 22 occasions (3 in the control, 14 in the depleted and 5 in the intact inhibition conditions), participants gave “X” as an answer. In addition, on 3 occasions (1 in each condition), they indicated that their mind was blank at the time of being stopped. This resulted in 452, 441, and 450 valid thought probes in the control, depleted and intact inhibition conditions, respectively.

Initially, all recorded thoughts were independently coded by the first author and a research assistant as either task-related or task-unrelated (cf. Plimpton et al. ,^2015^; Smallwood et al., 2003; Smallwood, Obonsawin, and Reid, 2003). Out of all valid 1343 thoughts, 1074 (80%) were task-unrelated thoughts (e.g., *meeting with my sister yesterday, going to the cinema after studying*), while 269 (20%) were classed as task-related thoughts (*this is so boring, don’t forget to push the button*) and were removed from further analysis. The agreement between the raters was 84%. Disagreements were solved by discussion. Second, out of 1074 task-unrelated thoughts, 188 (17.5%) were classed by participants as occurring deliberately rather than involuntarily. Since task-unrelated deliberate thoughts were not the primary focus of the present study (i.e., their occurrence would not depend on the depletion of a special inhibitory mechanism designed to keep involuntary thoughts at bay), they were excluded from further analyses, resulting in 886 spontaneous task-unrelated thoughts.

To examine the possible effects of depleting inhibitory control before the vigilance task on the number of involuntary task-unrelated thoughts reported during the vigilance task, the numbers of these thoughts in the control, depleted and intact inhibition conditions were entered into a one-way ANOVA with the condition as a between-subjects variable (see Table 3). The main effect of condition was not significant (*F*(2, 111) = 0.09, *p* = 0.914, *η*_*p*_^*2*^ = 0.01), indicating that spontaneous task-unrelated thoughts were reported with similar frequency across the three conditions.

**Table 3:** Mean numbers (and standard deviations) of different types of involuntary task-unrelated thoughts as a function of condition (control, intact inhibition, depleted inhibition)

	Condition					
	Control		Intact inhibition		Intact inhibition	
	M	SD	M	SD	M	SD
Total number of involuntary task-unrelated thoughts	7.82	2.60	7.87	3.02	7.63	1.95
Involuntary autobiographical memories (IAMs)	1.87	1.53	1.34	1.44	1.24	1.26
Involuntary future thoughts (IFTs)	1.63	1.40	1.29	1.18	1.71	1.80
Involuntary present-oriented thoughts	2.50	2.10	3.13	1.89	2.89	2.05
Involuntary atemporal thoughts	1.16	1.31	1.24	1.44	1.05	1.18

### Temporal focus of thoughts

After completing the vigilance task, participants were asked to decide whether each thought they reported was a memory of a past event, a future-oriented thought or something else.^7^ To ensure the thoughts had been categorized correctly, all the entries were also screened by the first author and the research assistant. All entries identified by participants as memories and future-oriented thoughts were in line with judgements by the judges. However, some of the thoughts classed as autobiographical memories or future thoughts by the judges were not identified as such by participants.^8^ Re-evaluated entries (e.g., *the first time I went to Netherlands, having a meeting tomorrow morning*) only with an agreement of 100% between judges were included in the analysis.^9^

Since the main focus of the present paper was on IFTs and IAMs, they were entered into a 3 condition (control, depleted inhibition, intact inhibition) × 2 temporal focus (past, future) mixed ANOVA with repeated measures on the last factor (for means see Table 3). Neither the main effects of condition [*F*(2, 111) = 1.79, *p* = 0.172, *η*_*p*_^*2*^ = 0.03], the temporal focus of thought [*F*(1, 111) = 0.10, *p* = 0.752, *η*_*p*_^*2*^ = 0.01], nor the condition by temporal focus interaction [*F*(2, 111) = 1.20, *p* = 0.304, *η*_*p*_^*2*^ = 0.02], were significant.^10^ For the sake of completeness, we repeated the above analysis by including the numbers of present-oriented and atemporal thoughts into a 3 (condition) by 4 (temporal focus) mixed ANOVA. Neither the main effect of condition or the condition by temporal focus interaction were significant (*Fs* < 1.31, ps > 0.253). However, there was a significant main effect of the temporal focus of thought [*F*(3, 333) = 24.193, *p* = 0.001, *η*_*p*_^*2*^ = 0.18], showing the highest number of present-oriented thoughts compared to the other types of thoughts (see Table 3).

To evaluate the possibility that the depletion manipulation did not last throughout the entire vigilance task, and had an effect on the number of spontaneous thoughts only during the first half of the vigilance task, we repeated the above analysis by including the additional within-subjects variable of the vigilance task phase (1st half, 2nd half). However, the 3 condition (control, intact, depleted) x 2 temporal focus (past, future) x vigilance task (1st half, 2nd half) mixed ANOVA with repeated measures on the last two factors did not result in any effects of condition, either alone or in interaction with vigilance task phase, all *p* > 0.13. Furthermore, we repeated this analysis including past and future thoughts from only the first four and last four thought probes in the vigilance task. Again, no significant effects were obtained, all *p* > 0.179. In summary, the results of these additional analyses suggest that the effect of depletion manipulation (or rather, its absence) was stable across the time, and comparable both at the beginning and at the end of the vigilance task.

### Correlations between ratings of fatigue and task-unrelated involuntary thoughts

Some previous studies have reported positive associations between increased cognitive fatigue and levels of mind-wandering (e.g., McVay & Kane, 2009, 2010; Zhang & Kumada ,^2017^). To investigate this relationship in our sample, we calculated correlations between the ratings of perceived fatigue (i.e., general, physical and mental tiredness) and the number of IFTs, IAMs, present-oriented and atemporal thoughts. Given that the three conditions did not differ in the number of these thoughts, the correlations were calculated on the data pooled across the three conditions. None of the correlations between the different ratings of fatigue and types of thoughts reported were significant (all *p* > 0.309).

## Discussion

We investigated the role of cognitive inhibition on the frequency of IFTs and IAMs. Participants were randomly assigned to the depleted inhibition, intact inhibition and the control conditions. By having participants in the depleted and intact inhibition conditions perform incongruent and congruent Stroop tasks, respectively, before completing the vigilance task, we were able to manipulate the level of available cognitive inhibition resources in these two conditions. In addition, we made every attempt to keep the conditions comparable in terms of several other background variables (cognitive and emotional). In line with the cognitive inhibition dependency hypothesis, it was predicted that the frequency of IAMs and IFTs would increase in the depleted inhibition condition in comparison to the intact inhibition and the control conditions. However, in contrast to the prediction, the number of IFTs and IAMs did not differ across the experimental and control conditions during the subsequent vigilance task. To evaluate the possibility that the depleted inhibition affected the number of IFTs and IAMs only at the earlier phases of the vigilance task, the additional analyses were conducted on the number of involuntary thoughts in the first half and the first third of the vigilance task, but they also resulted in non-significant findings.

Another interesting and novel finding that emerged from our study, concerns possible effects of fatigue on the number of reported IAMs and IFTs. Thus, participants in the intact inhibition condition reported significantly higher levels of general, physical and mental fatigue than participants in the control condition. These increased levels of self-reported fatigue resulted from the prolonged performance of the congruent Stroop task that required sustained cognitive effort, and was reflected in actual performance decrements in the vigilance task in terms of increased response time to the target stimuli (see Table 1). The fact that the response times in the vigilance task were shorter in the control condition compared to other two experimental conditions lends additional support to the notion that attentional resources were indeed reduced in these ‘fatiguing’ conditions. Despite these differences in self-reported fatigue, participants in the control and intact inhibition conditions did not differ in the number of reported IFTs and IAMs. In addition, correlations between the ratings of fatigue and the number of reported IFTs and IAMs in the entire sample were not significant. Below, we will first discuss the implications that these findings have for our understanding of the nature and underlying mechanisms of IAMs and IFTs, followed by a discussion of possible limitations and future avenues for research.

### Theoretical implications

Given that IFTs and IAMs are predominantly triggered by easily identifiable triggers, and the ubiquity of such triggers, the important theoretical question in the literature concerns the underlying mechanisms of such involuntary episodic MTT and why we are not constantly flooded by IAMs and IFTs in daily life. According to the influential model of autobiographical memory by Conway & Pleydell-Pearce (2000), autobiographical memories (and by extension, thoughts about possible future events) are constantly activated by incidental external and internal triggers, but the inhibitory control mechanism keeps suppressing these task-irrelevant thoughts, preventing them from reaching consciousness. In the present study, we investigated the existence of this hypothetical inhibitory mechanism using a well-established paradigm of depleting inhibitory control (Radel et al., 2015; Muraven & Baumeister, 2000; Hagger, Wood, Stiff, & Chatzisarantis, 2010), and assessing the number of IFTs and IAMs reported by participants after the depletion manipulation. The findings did not provide support for the existence of such a ubiquitous control mechanism.

One possible reason for this null effect is that the inhibitory control mechanism switches on only when people are engaged in attentionally demanding activities (e.g., reading, writing, having a conversation), because these are the activities that can be negatively affected by the occurrence of involuntary thoughts. If this is the case, the vigilance task used in the present study with its medium level of attentional demands (as reflected by mean ratings of concentration) would not be sensitive enough to examine the cognitive inhibition dependency hypothesis. Future studies could manipulate the difficulty level of the vigilance task to assess the idea that the depletion manipulation will increase the IFTs and IAMs during the difficult, but not the standard version of the vigilance task. Initial support for this idea comes from a study by Barzykowski & Niedźwieńska (2018a), who found that the proportion of IAMs (out of all the spontaneous task-unrelated thoughts reported) was higher in the attentionally highly demanding than in a standard vigilance task.

An alternative explanation is that the inhibitory control mechanism may not be necessary to keep the constantly activated involuntary episodic thoughts from coming to mind consciously. Indeed, our working memory has limited capacity and, at any given time, our mind is occupied by thoughts (either task-related or task-unrelated), which may prevent other thoughts from coming to mind simply because there is no extra capacity to experience these thoughts (cf. Kvavilashvili & Mandler, 2004). In other words, even if a particular IAM or IFT is ready to spring to one’s mind, if at that moment one’s mind is already attending to the environment or is engaged in other activities or thoughts, this may be sufficient to prevent these involuntary thoughts coming to one’s mind. For example, in a recent laboratory study by Floridou, Willimson, & Stewart (2017), participants watched two brief non-dialogue film trailers with music and then spent 5 min either doing nothing (eyes closed) or making a mark on a paper when seeing a blue dot on the screen (occurring in predictable alternate order with a red dot). Participants were then probed for the types of thoughts they had during the previous 5 min including any involuntary musical imagery (the tunes that they just had heard popping to their mind). While 65% of participants in the no task condition reported having involuntary musical imagery, this dropped to 32.5% in the group who were simply monitoring the blue dot. This finding suggests that even relatively undemanding activities, such as attending to predictable stimuli in the external environment, can substantially reduce the number of involuntary cognitions in one’s mind (for similar findings on IAMs, see Vannucci, Pelagatti, Hanczakowski and Chiorri, 2018).

Another possible mechanism for why we are not flooded by IAMs and IFTs, proposed by Berntsen, Staugaard, and Sørensen (2013), refers to the cue overload. According to the cue overload principle, a particular cue may be associatively related to more than one past event (Berntsen, 2009). Therefore, the more events are associated with a particular cue, the less efficient this cue will be in triggering any of them. In line with this, Berntsen et al. (2013) demonstrated that a particular sound was a less effective cue for triggering an involuntary memory of a visual scene if, at encoding, it was also associated to several other visual scenes. Because a lot of cues that we encounter on a daily basis (people, objects, places, sounds, etc.) are associatively linked to several or many possible past or future events, this may prevent us from being flooded by IAMs and IFTs as they are less likely to be formed in response to such cues. Taken together, it appears that several variables can influence the occurrence of IFTs and IAMs in addition to, or instead of, the putative inhibitory control mechanism. The results of the present study are important, because they show that the role of the inhibitory mechanism is perhaps not as strong as suggested by the inhibitory control dependency hypothesis.

Furthermore, findings in relation to mental and physical fatigue provide novel insights into the underlying mechanisms of IAMs and IFTs. Given that mental fatigue has been shown to lead to sub-optimal performance and increased errors in tasks relying on executive control and strategic retrieval processes (e.g., Van der Linden, Frese, & Meijman, 2003; Lorist, 2008), our results provide further support to the idea that involuntary episodic past and future thoughts are mediated by more automatic processes that do not require effortful retrieval or constructive processes (cf. Berntsen, 2010). This finding accords well with the results obtained by Cole et al. (2016) showing comparable and rapid retrieval latencies for both IFTs and IAMs in response to incidental cues on the screen. The findings are particularly important for the newly emerging field of research on IFTs, as they suggest that significant differences may exist in involuntary and voluntary episodic future MTT. Although voluntary forms of episodic future simulations rely on constructive and effortful process, the present results provide new evidence that the occurrence of IFTs may be less reliant on such executive control processes (cf. Cole et al., 2016).

### Possible limitations and future avenues for research

While our results suggest that the occurrence of IFTs and IAMs may not depend on inhibitory control, there is a question whether any aspect of our procedure imposes constraints on this interpretation. First, it can be argued that the manipulation used to deplete cognitive inhibition resources was inefficient, which would explain a lack of expected differences between the conditions in the number of involuntary mental contents. However, the majority of previous studies on the effects of depletion on self-control have used fairly brief depletion tasks (about 5–10 min) and have not verified the presence of depletion at the end of the manipulation with another task measuring inhibitory control (for meta-analysis, see Hagger et al., 2010). In the present study, we used an even stronger version of the depletion manipulation (i.e., longer and with higher proportion of incongruent trials) than the one that was successfully used by Radel et al. (2015) with a 40-min long depletion task and 50% incongruent trials. In addition, our manipulation successfully impaired inhibitory control in participants who performed the incongruent Stroop Task, before they started the vigilance task. This should have resulted in differences between experimental conditions if inhibitory control played an important role in the occurrence of IFTs and IAMs.

Second, one may question whether the depletion of inhibition, confirmed by the Simon task immediately after the Stroop task, lasted long enough to have noticeable effects on IAMs and IFTs during the subsequent vigilance task. In addition, there was up to an 8 min delay between the Simon task and the vigilance task, in which participants completed the PANAS and answered questions about fatigue, motivation and concentration. According to the strength model of self-control (Muraven& Baumeister, 2000), cognitive resources are limited and a period of rest should lead to the replenishment of the depleted resource. However, to the best of our knowledge, there has been very little research on the time needed to restore the depleted inhibitory control resources. For example, Tyler & Burns (2008) demonstrated that the depletion effect (resulting from a 6-min long mental and physical exercise) on a subsequent self-control task (persistence on a handgrip squeeze test) was eliminated with a 10-min delay (filled with questionnaires) between the tasks, but not with a 1-min and 3-min delay intervals. However, Oaten, Williams, Jones and Zadro (2008), who used a different depletion manipulation (a 5-min computer game creating the impression of being socially ostracised), found that the negative effects of depletion (measured by the amount of food or drink consumed), were present even after a 45-min delay (filled with questionnaires). Moreover, a meta-analysis by Hagger et al. (2010) showed that depletion effects were stronger in studies that used delays filled with questionnaires and manipulation checks than in the studies without delays between a depletion and a subsequent self-control task. Based on this evidence, we can be fairly certain that the depleted inhibitory control was not restored in our study by the time participants started the vigilance task.

It is also less likely that a full recovery of inhibitory resources occurred during the ongoing vigilance task. We addressed this issue by examining the number of IFTs and IAMs in the initial phases of the vigilance task, but they were not different from the numbers reported later in the task. Moreover, since the vigilance task induced boredom, and required medium to high levels of concentration and effort (see Table 3), it may be considered as a depleting task itself (Warm, Parasuraman, & Matthews, 2008), which further reduces the possibility that the resources depleted by a 60-min incongruent Stroop task were fully restored during the vigilance task.

The third, and related, question concerns the domain generality/specificity of inhibitory and cognitive control, and the possible absence of transfer from one domain to another. In other words, it is possible that the reduction in the capacity to exert inhibitory control in the context of the Stroop task did not affect the inhibitory mechanism involved in the occurrence of IFTs and IAMs. However, many studies using the sequential-tasks paradigm, have already shown that the performance on the first inhibitory control task, such as the Stroop task, can influence the performance in a variety of other subsequent tasks, such as the physical effort maintenance (Pageaux, Lepers, Dietz, & Marcora, 2014) or decision making (Yam, Chen, & Reynolds, 2014). Importantly, the presence of such transfer effects from an initial inhibitory control task to a subsequent task has been confirmed in a recent meta-analysis by Dang (2017), which showed significant depletion effects after performing the Stroop task with a moderate effect size (i.e., Hedge’s *g* = 0.44, 95% CI = 0.18–0.69). Consequently, if the occurrence of involuntary mental contents was limited by the inhibitory control, we should have been observed it in the present study.

Finally, we used the probe-caught method in which participants were randomly stopped on 12 occasions during the vigilance task and asked what was going through their mind at the exact moment they were stopped (see Plimpton et al., 2015). One could argue that the probe-caught method may not be as sensitive in detecting the effects of depleted inhibition as the self-caught method, in which participants stop themselves to report the occurrence of spontaneous thoughts (e.g., Barzykowski & Staugaard, 2016, 2018; Barzykowski & Niedźwieńska, 2016, 2018a, 2018b; Schlagman & Kvavilashvili, 2008; Cole et al., 2016; Vannucci et al., 2014), because participants in the depleted inhibition condition could have been experiencing increased frequency of IFTs and IAMs in time periods between the two consecutive stops. However, the probe-caught method was specifically chosen to obtain unbiased measures of the depletion effect, because in the self-caught method, the depleted resources could also negatively affect participants’ monitoring or meta-awareness for the occurrence of IFTs and IAMs (e.g., see Vannucci et al., 2018). In addition, our participants were stopped fairly frequently during the vigilance task (on average once every 83 s), and on the majority of the stops, they reported task-unrelated spontaneous thoughts. Therefore, this should have given us a reasonably good chance of detecting effects of depletion if they were present.

### Final conclusions and future directions

While we used a long and robust experimental manipulation aimed at depleting the cognitive resources needed for inhibition, we were not able to observe a significant increase in the retrieval of involuntary mental contents in general, and IFTs and IAMs, in particular. Our findings demonstrate that the impaired inhibition may not affect the occurrence of such thoughts, and therefore, they do not support the idea that involuntary mental contents rely strongly on the special inhibitory control mechanism. However, results of the present study raise interesting questions for future research about the underlying mechanisms of involuntary episodic MTT, by studying the role of inhibition in combination with other potentially important variables such as ongoing task difficulty and the nature of incidental triggers in the environment.

Another interesting avenue for research is to adopt an individual differences approach and pre-select participants based on their performance scores on several different conflict tasks requiring high levels of inhibitory control. It is possible that participants with high levels of inhibitory control report fewer IFTs and IAMs in the laboratory vigilance task used in the present study, than participants with low inhibitory control abilities. Alternatively, one could investigate a clinical group of adults with impaired cognitive control. For example, low inhibition efficiency is a characteristic symptom of ADHD (Barkley, 1997) and schizophrenia (Beech, Powell, McWilliam, & Claridge, 1989), and several studies on individuals diagnosed with ADHD (e.g., Seli et al. 2015; see also; Jonkman et al., 2017) and schizophrenia (e.g., Elua, Laws, & Kvavilashvili, 2015) have demonstrated elevated levels of spontaneous mind-wandering in these individuals. It is therefore highly likely that they may be also more susceptible to experiencing IFTs and IAMs compared to non-clinical populations. We are currently exploring some of these possibilities in follow-up studies, and believe that this research may ultimately provide interesting insights into cognitive mechanisms of involuntary episodic MTT.

## Electronic supplementary material

Below is the link to the electronic supplementary material.

Supplementary material 1 (DOCX 14 KB)

## Footnotes

1 In this task (Simon, 1969), participants have to decide between a left- and right-hand key presses according to the colour of a visual stimulus. This target is presented either to the left or to the right of a central fixation point. In the congruent trial, required response corresponds spatially to the stimulus location. In the incongruent trial, required response does not correspond (the location is irrelevant). Therefore, the “Simon effect” occurs when the location automatically triggers a response impulse while the relevant colour needs to be converted into the required response in accordance to the given instruction (Suarez et al., 2015). While in congruent stimulus–response associations, both the irrelevant location and the colour activate the required response, in incongruent associations the irrelevant location triggers the wrong response.

2 In this task (Eriksen & Eriksen, 1974), participants have to press the arrow keys with either the left or right index finger according to the direction pointed by the centre target arrow. This target arrow is surrounded by other arrows (flankers) that are to be ignored. In congruent trials all targets, including flankers, indicate the same response (they are pointing the same direction). In incongruent trials, they indicate the opposite directions and flankers activate wrong automatic response that is to be ignored and inhibited.

3 The presentation time of each trial was increased to keep the mean interval between the thought probes (83 s) comparable to the mean interval in the Plimpton et al. (2015) study (81 s), who used the larger number of trials (600 instead of 400).

4 The Polish adaptation of verbal phrases is described in detail elsewhere (Barzykowski & Niedźwieńska, 2016, p. 6). Briefly, they were rated by 12 independent coders as positive, neutral and negative.

5 By employing two practice sessions, we were able to sequentially provide participants with crucial elements of the procedure (i.e., instructions for the vigilance task only, followed by instructions how to record involuntary thoughts during the thought probes).

6 According to Whelan (2008), very slow reaction times, due to the subject’s inattention, can strongly influence the outcome of hypothesis tests, and should be excluded from the analyses. Ratcliff (1993) argues that, eliminating response times above an absolute cutoff point is the most appropriate method for maintaining the highest power. In our study, the cutoff points of 100 and 1300 ms were chosen according to the outlier labelling rule by Hoaglin, Iglewitz and Tuckey (1986). Specifically, the response times that were 2.2 times “the interquartile range below the bottom or above the upper quartile value of the distribution” (Marwerde 2017, p. 135) were treated as outliers (Hoaglin & Iglewicz, 1987).

7 This category included thoughts classed as present thoughts (*I have dry eyes, I am hungry*) and atemporal thoughts (*Hegel was an awesome philosopher*).

8 As pointed out by Barzykowski & Niedźwieńska (2018a, p. 123), due to the automated nature of the task, the decision whether a mental content was or was not a memory was irreversible, and this could have resulted in some errors that the participants committed in the categorization task.

9 In total, there were 170 entries re-evaluated from which 73% were included in the analysis and 27% were inconclusive. In total, the re-evaluated entries constituted 19% of entries included into the analysis.

10 Although it may be argued that the nature of the data may require statistical analysis basing on a Poisson rather than Gaussian distribution (as it is recommended for count-valued data; e.g., Inouye, Yang, Allen, & Ravikumar, 2017), we used the ANOVA model which is rather robust to violations of normality (Field, 2009). However, this main analysis was repeated using the generalized estimating equations (GEE) basing on a Poisson distribution. The GEE model demonstrated similar results to the ANOVA model; namely, neither the main effects of condition (*χ²*(2) = 3.67, *p* = .16), the temporal focus of thought (*χ²*(1) = 0.15, *p* = .70), nor the condition by temporal focus interaction (*χ²*(2) = 2.13, *p* = .34), were significant.
