# Supplementary material for: Why are we not flooded by involuntary thoughts about the past and future? Testing the cognitive inhibition dependency hypothesis
Source: Psychol Res. 2018 Nov 27;83(4):666–83. doi: 10.1007/s00426-018-1120-6 (PMC6529375; doi:10.1007/s00426-018-1120-6)
Supplement: Supplementary file 1 — Supplementary material 1 (DOCX 14 KB) [file 426_2018_1120_MOESM1_ESM.docx]

**Appendix**

Written verbal instructions provided to participants via computer program during the experimental session

The goal of this task is to study people’s ability to concentrate long on relatively monotonous and repetitive stimuli. More precisely, we are interested in fluctuations in concentration and thought during such monotonous tasks. Therefore, you will be shown a large number of different patterns of either horizontal or vertical lines on a computer screen. Each time you see vertical lines on the screen please press the red button on the keyboard. Apart from the lines there will be also some word phrases displayed in the center of the screen. The condition you are taking part in addresses the ability to concentrate on relatively monotonous stimuli regardless of the fact that words and phrases are displayed at the same time. In another condition, participants have to concentrate on the words and phrases regardless of the fact that patterns of lines are displayed at the same time. For your better understanding of the task you are about to perform a short practice trial. Remember, each time you see vertical lines, press the red button on the keyboard.

*[After completing the trial session]*

As you can see, this experiment is about people’s attention and their concentration during fairly lengthy monotonous tasks. You might be familiar with the situation in which your thoughts wander off during an easy monotonous task (for example, driving). However, at critical points, such as when approaching roundabouts, you will need to pay attention to what you are doing again. Our study is interested in these fluctuations in concentration and thought during such monotonous tasks. The main vigilance task is similar to the practice one but longer. In addition, the presentation will occasionally stop, and you will be prompted to record your concentration level and thoughts at the moment you were stopped. As you can see, although this task is quite simple, it can be difficult to maintain concentration. As with the driving example, your thoughts may drift to matters unrelated to the task. These thoughts can be about anything, including: personal goals, words, random associations, current concerns, the past, present or the future. They may be specific or general thoughts that pop into your mind spontaneously, or they may be something you have deliberately chosen to think about. It doesn’t matter if your concentration and thoughts fluctuate in this way throughout the ﻿presentation, but please ensure that you write down the content of your thoughts at the exact moment you are stopped. Each time you are stopped by the presentation, you will be provided with a questionnaire to record your thoughts and assess your level of concentration. It is not important what it will be and how interesting you will find your thought. You can refrain from reporting particularly sensitive thoughts by typing “X” as an answer or (if possible) by providing a general description of your thought rather than a detailed account.
